# Peritoneal dialysis peritonitis by anaerobic pathogens: a retrospective case series

**DOI:** 10.1186/1471-2369-14-111

**Published:** 2013-05-24

**Authors:** Chia-Ter Chao, Szu-Ying Lee, Wei-Shun Yang, Huei-Wen Chen, Cheng-Chung Fang, Chung-Jen Yen, Chih-Kang Chiang, Kuan-Yu Hung, Jenq-Wen Huang

**Affiliations:** 1 Department of Traumatology, National Taiwan University Hospital, Taipei, Taiwan; 2 Division of Nephrology, Department of Internal Medicine, National Taiwan University Hospital, Taipei, Taiwan; 3 Division of Nephrology, Department of Internal Medicine, National Taiwan University Hospital Yun-Lin Branch, Yun-Lin County, Taiwan; 4 Division of Nephrology, Department of Internal Medicine, National Taiwan University Hospital Hsin-Chu Branch, Hsin-Chu County, Taiwan; 5 Department of Emergency Medicine, National Taiwan University Hospital, Taipei, Taiwan; 6 Department of Geriatrics and Gerontology, National Taiwan University Hospital, Taipei, Taiwan; 7 Department of Integrated Diagnostics and Therapeutics, National Taiwan University Hospital, 7 Chung-Shan South Road, Taipei, 100, Taiwan

**Keywords:** Anaerobics, End-stage renal disease, Peritoneal dialysis, Peritonitis

## Abstract

**Background:**

Bacterial infections account for most peritoneal dialysis (PD)-associated peritonitis episodes. However, anaerobic PD peritonitis is extremely rare and intuitively associated with intra-abdominal lesions. In this study, we examined the clinical characteristics of PD patients who developed anaerobic peritonitis.

**Methods:**

We retrospectively identified all anaerobic PD peritonitis episodes from a prospectively collected PD registry at a single center between 1990 and 2010. Only patients receiving more than 3 months of PD were enrolled. We analyzed clinical features as well as outcomes of anaerobic PD peritonitis patients.

**Results:**

Among 6 patients, 10 episodes of PD-associated peritonitis were caused by anaerobic pathogens (1.59% of all peritonitis episodes during study the period), in which the cultures from 5 episodes had mixed growth. *Bacteroides fragilis* was the most common species identified (4 isolates). Only 3 episodes were associated with gastrointestinal lesions, and 4 episodes were related to a break in sterility during exchange procedures. All anaerobic pathogens were susceptible to clindamycin and metronidazole, but penicillin resistance was noted in 4 isolates. Ampicillin/sulbactam resistance was found in 2 isolates. In 5 episodes, a primary response was achieved using the first-generation cephalosporin and ceftazidime or aminoglycoside. In 3 episodes, the first-generation cephalosporin was replaced with aminoglycosides. Tenckhoff catheter removal was necessary in 2 episodes. Only one episode ended with mortality (due to a perforated bowel).

**Conclusion:**

Anaerobic PD-associated peritonitis might be predominantly caused by contamination, rather than intra-abdominal events. Half of anaerobic PD-associated peritonitis episodes had polymicrobial growth. The overall outcome of anaerobic peritonitis is fair, with a high catheter survival rate.

## Background

Peritoneal dialysis (PD)-associated peritonitis is an important contributor to morbidity and mortality, accounting for one-fifth to one-third of technique failures in PD practice [1,2]. Among all, Gram-positive bacteria constitute the most frequently isolated pathogens (45–65%), followed by Gram-negative bacteria (25–40%) and fungi (3–6%) [1-4]. Infection with mycobacteria and anaerobic bacteria is rare, both of which are found in <0.5% of peritonitis episodes [1].

Although anaerobic bacteria significantly outnumber other Gram-negative bacteria in the normal flora of the gastrointestinal (GI) tract, they have surprisingly weak pathogenicity as compared with other species [5]. Consequently, researchers place less focus on anaerobic bacteria. Furthermore, complicated methods for culturing these bacteria hamper the accurate identification of these pathogens [6]. However, anaerobic bacteria assume an increasingly important role by causing infections among immunocompromised hosts, including end-stage renal disease (ESRD) patients on dialysis [7].

ESRD patients acquire anaerobic infections mostly from hematogenous spread of GI sources or rarely, from dentition defects [5,8-10], but these scenarios are common only in those receiving hemodialysis. Anaerobic infections in PD patients are relatively rare, mostly encountered during PD peritonitis. According to the International Society for Peritoneal Dialysis (ISPD) guidelines, anaerobic PD-associated peritonitis episodes are discussed mainly within the polymicrobial peritonitis section, and surgical interventions are recommended if anaerobic pathogens coexist with other enteric microorganisms [11-13]. However, not all anaerobic PD-associated peritonitis episodes occur with intra-abdominal catastrophe. Issues pertaining to peritonitis from a single anaerobic pathogen also remain unexplored, and reports on anaerobic PD-associated peritonitis are extremely sparse. Therefore, the current study analyzed the clinical features and outcomes of anaerobic PD-associated peritonitis in our center.

## Methods

### Study design and setting

This was a retrospective analysis of a prospectively collected cohort in the National Taiwan University Hospital (NTUH) PD program. In our PD registry, all patients with ESRD initiated on PD or initially on hemodialysis but later switched to PD in our institute were identified, and entered into our PD registry. Only patients under maintenance PD for more than 3 months were eligible in the current study. We identified all episodes of culture-confirmed anaerobic peritonitis between 1990 and 2010. Peritonitis was diagnosed according to the presence of peritoneal signs and cloudy effluent with leukocyte counts >100/μL and >50% neutrophils. Relapse peritonitis was defined as peritonitis recurring within 4 weeks after the treatment of previous episodes involving the same pathogens, while repeat peritonitis was defined as peritonitis recurring after 4 weeks of previous episodes involving the same pathogens [11,12].

### Clinical data collection

We reviewed all PD patients’ demographic profiles, which included age, gender, and comorbidities such as diabetes mellitus (DM), hypertension, coronary artery disease (CAD), congestive heart failure (CHF), peripheral artery occlusive disease, and previous malignancy. Past experiences of admission and surgeries were also recorded.

For each anaerobic peritonitis episode, we recorded the PD vintage, modality (continuous ambulatory peritoneal dialysis [CAPD] or automated peritoneal dialysis [APD]), initial symptoms, most recent serum biochemistry profiles, blood leukocyte counts/C-reactive protein (CRP) levels during peritonitis episodes, pathogens identification results (cultures and/or Gram stain), and antibiograms. The presumed etiology of peritonitis included a break in sterility during the exchange procedures, GI flora translocation, major intra-abdominal events (including diverticulitis or perforated viscera), and undetermined. Patients with peritonitis were initially treated with empirical intraperitoneal cefazolin/aminoglycoside or cefazolin/ceftazidime (after 1998, due to concerns of aminoglycoside impairing residual renal function) according to ISPD recommendations [11-13], unless otherwise clinically indicated.

### Outcome variables

Outcome measures included primary response, antibiotic switch (secondary response), relapse peritonitis, repeat peritonitis, Tenckhoff catheter removal, and mortality. The primary response was defined as symptomatic improvement with effluent leukocyte counts <100/μL within 3 days under the first-line antibiotics. The secondary response was defined as a response to next-line antibiotics when effluent failed to clear with the first-line antibiotics [11-13].

### Ethical considerations

The ethics committee of the NTUH approved this study (NO. 201212165RINC). The local institutional review board did not mandate patient consent, since no interventions were performed, and patient privacy was not breached.

## Results

During the study period, a total of 328 patients received PD therapy in our institute for 35,211 patient-months, and the overall peritonitis rate was 1 per 56.2 patient-months. Among the 627 episodes of PD peritonitis, 6 patients developed 10 episodes caused by anaerobic pathogens (1.59% of all episodes). One patient developed 4 episodes within 3 years separately; another developed 2 episodes within 4 years; and the remaining patients each had 1 episode.

### Clinical features of patients with anaerobic PD peritonitis

Among these patients who developed anaerobic PD peritonitis, two-thirds of them were women, and the mean age was 46 years (range, 20–68 years). The mean patient vintage at the time of anaerobic PD peritonitis was 50 months (range, 5–109 months). Their comorbidities were as follows: five patients (83%) had hypertension, three patients (50%) had CHF and coronary artery disease (CAD), one patient (17%) had systemic lupus erythematosus and was receiving azathioprine; still another (17%) had a history of peptic ulcers. Past admission history included cystoscopy examination (one patient; 17%) and lupus flare-up (one patient; 17%). No patient ever received intra-abdominal surgery before. The most common primary causes of ESRD were chronic glomerulonephritis (3 patients), followed by DM (1 patient), lupus nephritis (1 patient), and Chinese herb nephropathy (1 patient).

One patient was receiving intravenous cefepime for hospital-acquired pneumonia before index peritonitis, while 2 patients were given intraperitoneal vancomycin and ceftazidime for preceding PD peritonitis weeks ago. No patient had recently undergone GI endoscopy. The most recent serum laboratory profiles before peritonitis (within one month) were provided in Table 1.

**Table 1:** The most recent serum biochemical profiles of patients before anaerobic PD peritonitis episodes

***Variables***	***Average***	***Range***
Hemoglobin (g/dL)	9.8	7.7-11.4
Albumin (mg/dL)	3.6	2.4-4.7
Creatinine (mg/dL)	9.0	5.7-12.3
Total cholesterol (mg/dL)	218	104-296

### Clinical courses of anaerobic PD peritonitis

The most common presenting symptoms were abdominal pain (8 episodes), followed by rebounding tenderness (6 episodes), fever (4 episodes), nausea/vomiting (3 episodes), diarrhea (1 episode), and loss of consciousness (1 episode). All patients had turbid dialysate. The initial blood leukocyte levels were 10,245/μL (range 5850–19,980/μL), with 84.2% neutrophils. The average CRP levels at presentation were 10.5 (range 0.61-29.7 mg/dL). Mean effluent leukocyte counts were 1978/μL (range 100–9000/μL), with 83.3% neutrophils.

Effluent Gram stain only identified Gram positive bacilli in one episode. Effluent cultures revealed *Bacteroides fragilis* in 4 episodes, *Lactobacillus* species in 3 episodes, *Bacteroides thetaiotaomicron* in 2 episodes, and *Peptostreptococcus* species in 1 episode. Half of the episodes showed polymicrobial growth, in which *Bacteroides* and *Lactobacillus* species were present in 4 and 1 episodes of mixed growth, respectively. The other organisms cultured were *Enterococcus* species, *Klebsiella pneumoniae*, and *Pseudomonas aeruginosa* as well as *Candida albicans*. None of them had concomitant bacteremia, and no patient had concomitant extra-gastrointestinal infection foci (including respiratory tracts).

Contrary to previous reports [11,12], the most common cause of anaerobic peritonitis was associated with sterility break (4 cases with a relevant history of contamination during exchange procedures). Structural GI lesions were identified in 2 episodes. One had diverticulitis, and the other was suspected to have small bowel perforation. One case resulted from GI flora translocation presenting as diarrhea for days before peritonitis. No obvious explanations were discovered in the remaining 3 episodes of anaerobic peritonitis.

The antibiotic susceptibilities of the anaerobic pathogens are shown in Table 2. All anaerobic isolates were susceptible to metronidazole and clindamycin. The rate of resistance to penicillin was the highest (4 isolates), followed by cefmetazole (3 isolates) and flomoxef (2 isolates). Resistance to beta-lactam/beta-lactamase inhibitors (ampicillin/sulbactam) was present in 2 isolates. One isolate showed intermediate resistance to cefmetazole.

**Table 2:** Antibiotic susceptibility of anaerobic pathogens of peritonitis episodes (n = 10)

***Antibiotics***	***Susceptible***	***Intermediate***	***Resistant***	**Not tested**
Cefmetazole	4	1	2	3
Chloramphenicol	7	0	0	3
Clindamycin	7	0	0	3
Flomoxef	5	0	2	3
Metronidazole	7	0	0	3
Penicillin	3	0	4	3
Ampicillin/sulbactam	5	1	1	3

### Clinical outcomes of anaerobic PD peritonitis

Table 3 displays the outcomes of patients with anaerobic PD peritonitis. For the first-line antibiotics, intraperitoneal cefazolin/ceftazidime was used in 5 episodes, while intraperitoneal cefazolin/tobramycin was used in 3 episodes (all before 1998). Two patients were given intravenous vancomycin/meropenem and imipenem/cilastatin with amphotericin B to cover potential nosocomial pathogens. A primary response was achieved in 5 episodes, and a secondary response occurred after antibiotic switching, from intraperitoneal cefazolin/ceftazidime to ceftazidime/amikacin or gentamicin in 3 episodes. Tenckhoff catheter removal was necessary in 2 patients because of refractory peritonitis, even under intravenous vancomycin/meropenem and imipenem/cilastatin. One patient was later switched to hemodialysis, and the other died of intestinal perforation 1 month later, considering that surgical intervention was unsuitable because of the patient’s poor condition. Those who responded to antibiotics were maintained on antibiotics for a total of 14 days. There was no relapse or repeat peritonitis after anaerobic PD peritonitis episodes.

**Table 3:** Clinical courses and outcomes of anaerobic peritonitis episodes (n = 10)

	***Offending pathogens***	***First-line antibiotics***	***Antibiotics switch***	***Outcomes***
Patient 1	*Bacteroides thetaiotaomicron*	IP cefazolin/ceftazidime	IP ceftazidime/amikacin	Cure
Patient 2	*Bacteroides fragilis*	IV vancomycin/meropenem		Technique failure with catheter loss
Patient 3	*Lactobacillus*	IP cefazolin/ceftazidime		Cure
	*Bacteroides thetaiotaomicron*	IP cefazolin/ceftazidime		Cure
	*Lactobacillus*	IP cefazolin/ceftazidime		Cure
	*Bacteroides fragilis*	IV imipenem/amphotericin B		Catheter loss; Mortality due to bowel perforation 1 month later
Patient 4	*Lactobacillus*	IP cefazolin/ceftazidime	IP ceftazidime/gentamicin	Cure
Patient 5	*Bacteroides fragilis*	IP cefazolin/tobramycin	IP ceftazidime/amikacin	Cure
	*Peptostreptococcus*	IP cefazolin/tobramycin		Cure
Patient 6	*Bacteroides fragilis*	IP cefazolin/tobramycin		Cure

Abbreviations: *IP *intraperitoneal; *IV *intravenous.

## Discussion

In the present case series, *Bacteroides* species were the most common anaerobic organisms identified from PD-associated peritonitis patients, and half of the episodes were accompanied by other enteric pathogens. Only 2 episodes were associated with proven GI lesions. All isolates showed 100% susceptibility to metronidazole and clindamycin. The overall outcome was fair with 80% antibiotic cure rate, 20% Tenckhoff catheter loss, and 10% mortality from index peritonitis episodes.

Anaerobic bacteria account for a substantial minority of PD-associated peritonitis episodes, and most reports on PD-associated peritonitis did not address this issue [14,15]. Therefore, a dedicated description of anaerobic peritonitis features is lacking in the literature. In the ANZDATA registry, anaerobic peritonitis accounted for only 0.1% of single-pathogen peritonitis over a 6-year period, rising to 1.1% if only polymicrobial episodes are considered [1]. In the United States and Canada, anaerobic peritonitis constitutes <1% of all episodes [2]. In our experience, anaerobic pathogens were causative in 1.59% of all peritonitis episodes, which was higher than other reports. A lower peritonitis incidence of other pathogens might underscore the percentage of anaerobic episodes in our PD patients. On the other hand, this might reflect the geographic or ethnic difference in host susceptibility to different pathogens, including anaerobes [16]. Nonetheless, the lower peritonitis incidence in our cohort suggests that our cases need to be interpreted in light of our institutional characteristics and the potential generalizability.

In the ISPD guidelines, anaerobic growth is frequently linked to intra-abdominal events requiring surgical attention [11,12] because delayed intervention could be detrimental [17,18]. However, in our case series, only 2 episodes had GI lesions identified. All 3 patients with GI-related etiologies had polymicrobial growth, and *Bacteroides* species were the main pathogens (3 episodes); while in contamination cases, *Lactobacillus* species were the predominant organisms isolated (3 episodes), followed by *Bacteroides thetaiotaomicron* (1 episode). In addition, one case of contamination also showed polymicrobial growth. From these data, it might be plausible that anaerobic peritonitis could be further divided into 2 categories, contamination-related or intra-abdominal event-related. Each might be featured by different anaerobic species (*Lactobacillus* vs. *Bacteroides*). Consequently, we postulate that neither the growth of anaerobic pathogens from effluent nor polymicrobial growth serves as a token for intra-abdominal events. It might be better to consider failure of technique sterility simultaneously to avoid unnecessary examinations or even operations.

In the previous reports of anaerobic infections, *Bacteroides* species were most commonly isolated, followed by *Clostridium* and *Peptostreptococcus* species [19,20]. Risk factors for anaerobic infection include male gender, advanced age, presence of cancer, DM, chronic obstructive pulmonary disease, liver diseases, heart diseases, and GI surgeries [19,21]. However, in our patients, only 10–20% had these predisposing factors. As we identified, a potential factor associated with anaerobic peritonitis in PD patients might be a recent exposure to antibiotics (3 episodes). It is then likely that PD patients with anaerobic peritonitis might bear different clinical features than the general population.

The choice of antibiotics in anaerobic PD-associated peritonitis has not been specifically addressed previously, and the ISPD guidelines have not included a section focused on anaerobic peritonitis [11,12]. For anaerobic pathogens, aminoglycosides have counteractive effects against earlier microbial pathogenicity, while clindamycin protects hosts from abscess formation [20]. The resistance rates for anaerobic pathogens among available reports are as follows: penicillin, approximately 30–60%; metronidazole, <5%; clindamycin, 6–38%; beta-lactam/beta-lactamase inhibitors, 5–15%; and cephamycin, 10–40% [5,19,20,22]. All the anaerobic isolates in our patients were susceptible to metronidazole and clindamycin, while 20–30% of cases were resistant to cephamycin and ampicillin/sulbactam. An interesting finding is that 50% of our cases responded well to cefazolin/ceftazidime, although these antibiotics are reported to have poor efficacy against anaerobes, while aminoglycosides retain their efficacy because of earlier use during disease courses, as stated above [5,20,23]. Several studies have demonstrated that empirical cefazolin/ceftazidime could be effective against mixed infections. These antibiotics could reverse the favorable local milieu for anaerobic growth provided by other Gram-negative aerobes [24,25]. From this view, 3 out of 5 polymicrobial anaerobic peritonitis episodes in our patients are responsive to cefazolin/ceftazidime use, and these antibiotics might exert an indirect effect of anti-anaerobics. Based upon our limited experience in the current case series, we proposed that first-line antibiotics with metronidazole or clindamycin could be rational for anaerobic PD peritonitis, especially for single anaerobic episodes. Past studies also concurred that clindamycin might be be useful for patients allergic to penicillin, with Gram-positive coverage requirements[26].

The presence of comorbidities and isolates of *Bacteroides*, *Clostridium*, or polymicrobial infections raises the risk of adverse outcomes in anaerobic infections [19,20]. In the ANZDATA registry, catheter removal was necessary in 100% of anaerobic peritonitis episodes, and 90% of cases required hospitalization [1]. However, their case number was only 5. Our findings suggested that the catheter outcome of anaerobic peritonitis was not as poor as previous studies showed (20% catheter loss; Table 3). This difference in outcome could be attributed to contamination as the most common cause of anaerobic peritonitis in this study, and early effective antibiotics could salvage the catheter. This was quite different from worse outcomes of patients with intra-abdominal events related to anaerobic peritonitis. Moreover, no repeat or relapse peritonitis episode was observed. In our limited experience, anaerobic PD-associated peritonitis patients showed a 10% mortality rate (Table 3). This high mortality rate, compared with <4% mortality with other pathogens [1,2], could be the result of intra-abdominal catastrophe rather than anaerobic peritonitis *per se*. Consequently, we suggest that anaerobic peritonitis might still carry a favorable outcome if no intra-abdominal events occur.

### Limitations

Our study had several limitations. First, the case number (6 patients, 10 episodes) was low, and could not permit detailed analysis of the clinical features and courses of anaerobic PD peritonitis. Second, our current experiences in managing anaerobic PD peritonitis were preliminary, and should not be considered the norm for these rare patients. Third, one patient in our series developed 4 times of anaerobic peritonitis, and thus might weigh more than other enrollees. Fourth, Chinese herb nephropathy is rare in Western countries, and whether this ESRD etiology could correlate with anaerobic peritonitis is unclear. Nonetheless, the fair outcome of these anaerobic PD peritonitis patients is interesting and warrants our attention. Further larger studies are needed to better guide clinicians in treating anaerobic PD peritonitis patients.

## Conclusion

Anaerobic peritonitis is very rare among all PD-associated peritonitis episodes, and *Bacteroides* species is the most common causative agent. Although polymicrobial infections occur in half of the episodes, contamination is still the single predominant etiology of anaerobic peritonitis. The overall outcome is fair if no intra-abdominal events occur, with only 20% of patients experiencing catheter loss.

## Competing interests

The authors have no relevant financial or non-financial competing interests to declare in relation to this manuscript.

## Authors’ contributions

Study design: CTC, HWC and JWH; Study conduct: CTC, HWC, SYL, WSY, CCF, CJW, CKC, KYH and JWH; Data collection: CTC, HWC, CKC, KYH and JWH; Data interpretation: CTC, HWC, SYL, WSY, CCF, CJW, CKC, KYH and JWH; Drafting: CTC, HWC, SYL, WSY, CCF, CJW, CKC, KYH and JWH; All authors read and approved the final manuscript.

## Pre-publication history

The pre-publication history for this paper can be accessed here:

http://www.biomedcentral.com/1471-2369/14/111/prepub
